# Revealing the High-Modulus Mechanism of Polyimide Films Prepared with 3,4′-ODA

**DOI:** 10.3390/polym13183175

**Published:** 2021-09-18

**Authors:** Li Zhu, Yinong Li, Shuhao Han, Hongqing Niu, Dezhen Wu, Shengli Qi

**Affiliations:** State Key Laboratory of Chemical Resource Engineering, Beijing University of Chemical Technology, Beijing 100029, China; 15910817966@163.com (L.Z.); li_yinong@foxmail.com (Y.L.); bucthanshuhao@163.com (S.H.)

**Keywords:** polyimide films, molecular dynamics, high modulus

## Abstract

To prepare PIs (polyimides) with desirable thermal and mechanical properties is highly demanded due to their widespread applications in flexible optoelectronic devices and printed circuit boards. Here, the PI films of BPDA/4,4′-ODA, BPDA/3,4′-ODA, PMDA/4,4′-ODA, PMDA/3,4′-ODA systems were prepared, and it was found that the PIs with 3,4′-ODA always exhibit a high modulus compared with the PIs with 4,4′-ODA. To disclose the mechanism of high-modulus PI films with 3,4′-ODA, amorphous PI models and uniaxial drawing PI models were established and calculated based on MD simulation. The PI structural deformations at different length scales, i.e., molecular chain cluster scale and repeat unit scale, under the same stress were detailed and analyzed, including the variation of chain conformation, bond length, bond angle, internal rotation energy, and torsion angle. The results indicate that PIs with 3,4-ODA have higher internal rotation energy and smaller deformation with the same stress, consistent with the high modulus.

## 1. Introduction

Polyimide (PI), originating from the polycondensation of diamine and dianhydride, is a kind of polymer possessing prominent mechanical properties, good temperature stability and a low coefficient of thermal expansion. Among its various structures, the PI based on the pyromellitic dianhydride (PMDA)/4,4′-oxidianiline (4,4′-ODA) system is widely used in microelectronics, engineering, and aerospace fields because of its simple synthesis process, low cost, and high performance [1,2,3,4]. For applying to different situations, polyimide could be prepared for diverse productions by varying the processing technique, including fibers, films, resin, and so on. During the age of electronic information, one significant application of polyimide films is the robust substrate for flexible optoelectronic devices and printed circuits [5], in which the PI films require both a high modulus to resist deformation and excellent thermal stability to withstand high temperatures during processing [6,7].

The traditional PI film is difficult to dissolve in organic solvents, so it is mainly prepared by a two-step method, that is, aromatic diamine and dianhydride are condensed and polymerized into the precursor of poly (amic acid) (PAA) in polar aprotic solvents, such as N-methyl-2-pyrrolidone (NMP), dimethylformamide (DMF), and dimethylacetamide (DMAc), and then dehydrated to form PI [8]. Therefore, the properties of PI depend on the type of diamine and dianhydride [9,10]. The different chemical structures of the two monomers could produce a great influence on the molecular chain conformation, condensed structure, and mechanical performance of PI [5,11,12].

In the process of designing and developing new materials, molecular simulation technology has been gradually established and widely used in biology, materials, and other fields [13,14]. Molecular dynamics (MD) simulations and Monte Carlo (MC) [15,16,17] techniques have been demonstrated to be highly consistent with experimental results. This technology has been utilized to investigate the various properties of PI [18,19,20,21,22]. For example, Lei et al. [23] used this method to analyze the multilevel structure of some polyimide film systems. Li et al. [24] calculated the gas permeability and selectivity in the study of PI hollow fiber membranes. Lin et al. [25] simulated and analyzed the orientation process of PI. The orientation model of PMDA/4,4′-ODA polyimide with different draw ratios was established by the uniaxial tensile method, and the transition of the condensed structure during the orientation process was analyzed. V. M. et al. [26] discussed the correlation between the high-temperature local mobility and the mechanical properties; they believed that the modulus and the rigidity of the molecular chain are related. However, compared with the 3,3′,4,4′-biphenyldianhydride (BPDA)/4,4′-ODA system with a flexible chain, the PMDA/4,4′-ODA system with a rigid chain exhibits weaker mechanical properties [27,28,29]. There is still a lack of further mechanism analysis of the effect of chemical structure on mechanical properties.

In order to analyze the influencing factors on the mechanical properties of polyimide from the mechanism, we have carried out many studies, such as an analysis of the chain conformation of PI [23], an analysis of rigidity and flexibility, an analysis of bond length and bond angle [25], and an analysis of deformation resistance and thermal stability [24].

In this paper, the PI films of BPDA/4,4′-ODA, BPDA/3,4′-ODA, PMDA/4,4′-ODA, PMDA/3,4′-ODA systems were prepared, and their mechanical and thermal properties were tested. The condensed state models of the four systems were established by MD simulation, and the corresponding orientation models of the four systems after uniaxial stretching were also built and studied. The multi-scale analysis was carried out based on molecular simulation. The change of covalent bond length and torsion angle barrier are discussed at small scales. On the scale of the polymer chain, the conformation was analyzed by comparing mean-square end-to-end distance, mean-square rotation radius, characteristic ratio, and torsion angle distribution. The performance change of polyimide film caused by chemical structure alteration was analyzed from the principle at various scales to reveal the effects of multistage conformation on multistage structure and properties. The prepared polyimide film provides a good application prospect in the field of flexible optoelectronic devices and printed circuit boards and guides the manufacture of high-performance polyimide products.

## 2. Materials and Methods

### 2.1. Materials

The monomer PMDA was purchased from Nanjing Longsha Co., Ltd. (Jiangsu, China). The monomer 4,4′-ODA and 3,4′-ODA were purchased from Beijing Sinmaya Chemicals Co., Ltd. and recrystallized in ethyl acetate prior to use. The monomer BPDA was purchased from Shi Jiazhuang Hai Li Chemical Company (Shijiazhuang, China) and used after sublimation. The solvent DMAC was purchased from Tianjin Fu Chen Chemicals Reagent Factory (Tianjin, China). The deionized water used in the experiment was prepared by the Laboratory Water Purification System (Beijing, China).

### 2.2. Preparation of PI Films

The molar ratio of PMDA and ODA was 1:1, ODA was dissolved in DMAc, and PMDA was added step by step. The PAA solution of 15 wt% PMDA/ODA system with appropriate viscosity was obtained by stirring at 0 °C for 6 h. After casting the PAA solution, the PAA solution was placed on the ultra-clean table for 48 h to eliminate foaming and ensure a uniform thickness of the film. After putting the film into a blast oven, the atmosphere was heated up to 300 °C according to a certain program, held for 2 h and slowly cooled.

The preparation process of the other three systems of PI films was the same as above.

### 2.3. Characterization

Fourier Transform Infrared Spectrometer (Nexus 670, Nicolet Company, Madison, WI, USA) measurements were performed with a scanning wavenumber ranging from 4000 cm^−1^ to 400 cm^−1^ at a scanning number of 16 in an ambient atmosphere. The FT-IR spectra from molecular simulation were calculated based on an optimized geometry at DFT/B3LYP/6-31G(d) level.

Dynamic thermomechanical analysis was employed on a DMA Q800 (TA Instruments, New Castle, DE, USA) at a temperature from 50 to 450 °C under an N_2_ atmosphere. The heating rate and load frequency were set as 5 °C/min and 1 Hz, respectively.

The modulus was obtained by a universal tensile testing machine. The film samples were cut to 4 mm × 20 mm and tested at 25 °C.

### 2.4. MD Simulations

In this work, four kinds of polyimide were selected for molecular simulations. These polyimides were derived from two kinds of dianhydride, i.e., PMDA, BPDA, and two isomeric kinds of diamine, i.e., 4,4′-ODA, 3,4′-ODA. The corresponding molecular models were constructed using Material Studio (MS) 8.0.

A molecular chain containing 20 repeat units [30,31,32] was constructed by the Homopolymer tool in MS. Cells with ten molecular chains were constructed for each system by the Amorphous Cell module in MS. The initial density of each box is 0.1 g/cc. After the geometry optimization of models, a model with minimum energy was selected for dynamic equilibrium with the COMPASS force field [16,33].

The dynamic equilibrium was divided into four parts [23,24,34]:

(i) Compress the cell by a 40 ps MD in the NPT ensemble (fixed number of particles, pressure, and temperature) at high pressure (0.5 GPa, approximate to the experimental density).
(ii) Anneal the cell by subsequent MD runs in the NVT ensemble (fixed number of particles, volume, and temperature) at 598 K and 298 K for about 40 ps.
(iii) Relax the cell by a 500 ps NPT MD and 0.0001 GPa to check the course of the density fluctuations.
(iv) Equilibrate the cell by a 1000 ps NVT MD run in order to further improve the equilibration.

The Nose thermostat (Q ratio of 1) and the Berendsen barostat (decay constant of 0.1 ps) were used in the MD simulation.

The tensile model was constructed by applying fixed stress to the amorphous model to make the model produce strain and ensure that the strain of the model was within the elastic strain range.

In order to analyze the conformation changes of molecular chains during uniaxial drafting, radial distribution function (RDF), mean-square displacement (MSD) length distribution, and torsion distribution were estimated using Forcite Analysis tools.

According to the covalent and non-covalent interactions, the possibility of finding another atom within a certain distance of one atom is calculated by using RDF, which can be used to calculate the bond length and other ordered distances [35]. The definition of RDF is per Equation (1):

(1) $$g\left(r\right)=\frac{n_{B}/4\pi r^{2}\Delta r}{N_{B}/V}$$

where $n_{B}$ is the number of *B* atoms at a distance of *r* in a shell of thickness Δ*r* from atom *A*, *N_B_* is the total number of *B* atoms in the system, and *V* is the total volume of the system.

MSD analysis was performed to determine the mode of displacement of particles followed over time [36]. The MSD is defined as Equation (2):

(2) $$<r^{2}\left(t\right)>=\frac{1}{N}\overset{N}{\underset{i=1}{\sum }}<{\left|r_{i}\left(t\right)-r_{i}\left(0\right)\right|}^{2}>$$

where $r_{i}\left(0\right)$ and $r_{i}\left(t\right)$ are the initial and final positions of molecules (mass centers of particle i) over the time interval *t*, *N* is the atom number, and brackets denote the ensemble average.

Fractional free volume (FFV) was calculated using the “Atom volume and surface” tool under the condition of applying a Connolly probe with 0 nm radius. The free volume is identified as the sum of static voids created by chain packing and transient gaps generated by thermal-induced chain rearrangement, which provides the diffusing molecules with a low-resistance path for transport. Internal free space and effective arrangement of molecular chains can be calculated by fractional free volume. FFV is given by Equation (3):

(3) $$\mathrm{FFV}=\frac{V-1.3V_{W}}{V}$$

In this equation, *V* and $V_{W}$ represent the polymer volume and the van der Waals volume, respectively.

Glass transition temperature (T_g_) is calculated by the density change of the model from high temperature to low temperature, step by step, at 25 K, and the step cooling method is closer to reality than the continuous cooling method [37], as T_g_ of stepwise cooling is closer to the experiment than continuous cooling [38].

The torsion angle is the dihedral angle of two conjugated planes, which can also be used to reflect the internal rotation of the polymer [39]. It is an important indicator of polymer conformation. Ψ1 is defined as the dihedral angle (C-N-C-C), and Ψ2 and Ψ3 are defined as the dihedral angle besides the ether linkage (C-O-C-C, C-C-O-C). The statistical torsion angle is 180 steps with 2° per step.

## 3. Results and Discussion

### 3.1. Model Validation Mechanical Properties of Polyimide and FT-IR

**Figure 1:**
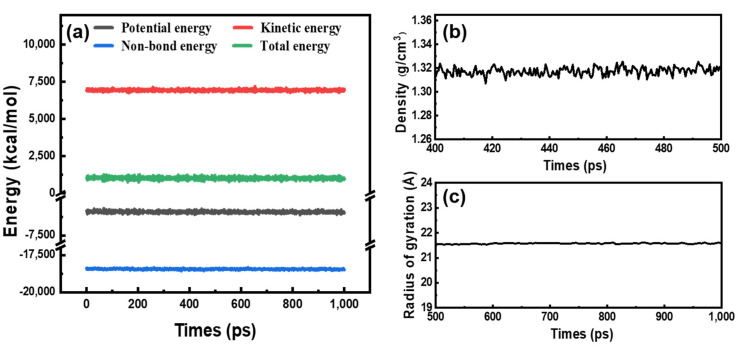
(**a**) Energy curves of the models; (**b**) density curve of the models; and (**c**) radius of gyration curve of the models (example is PMDA/4,4′-ODA).

To reveal the high-modulus mechanism of PIs with 3,4′-ODA in the present research, four types of polyimide models were obtained by binary condensation polymerization of PMDA, BPDA, 4,4′-ODA, and 3,4′-ODA were constructed for molecular modeling using Material Studio. The model validity and equilibrium check was performed by stating their density, energy, and gyration radius fluctuations during the long MD runs [40,41], as shown in Figure 1, by taking PMDA/4,4′-ODA systems as an example. The small density and energy fluctuations during the long MD runs can be clearly observed, and the gyration radius leveled off, demonstrating the stability of our molecular models.

**Figure 2:**
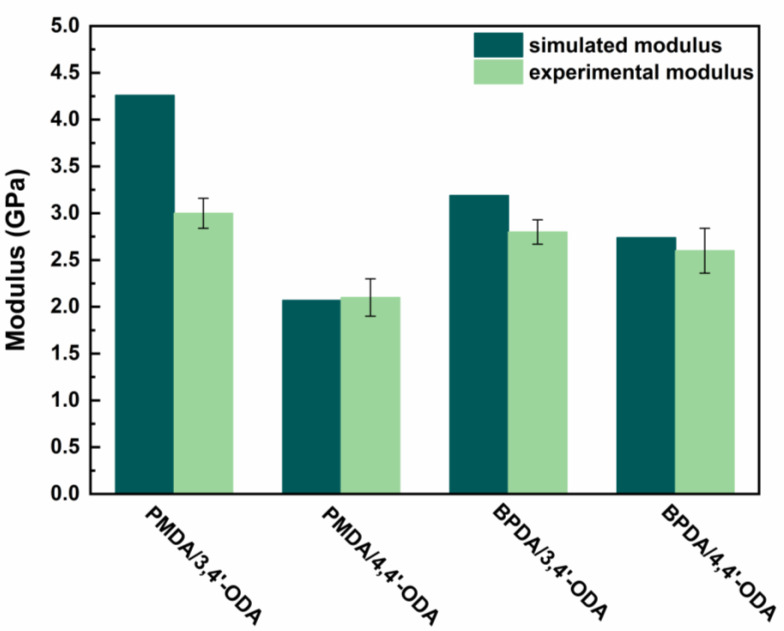
The modulus of different systems of polyimides.

As shown in Figure 2, the elastic modulus E was experimentally measured through the tensile test, while the modulus was simulated through the MD simulation. According to the results, the experimental modulus of the synthesized PIs, in descending order, is PMDA/3,4′-ODA (3.00 GPa) > BPDA/3,4′-ODA (2.80 GPa) > BPDA/4,4′-ODA (2.60 GPa) > PMDA/4,4′-ODA (2.10 GPa), indicating that the PIs with 3,4′-ODA exhibit a high modulus compared with the PIs with 4,4′-ODA. Similarly, the simulated modulus in descending order is PMDA/3,4′-ODA (4.26 GPa) > BPDA/3,4′-ODA (3.19 GPa) > BPDA/4,4′-ODA (2.74 GPa) > PMDA/4,4′-ODA (2.07 GPa), proving the rationality of our molecular models. Thus, the constructed models were used for further exploration of the properties and chain behaviors of the polyimides.

**Figure 3:**
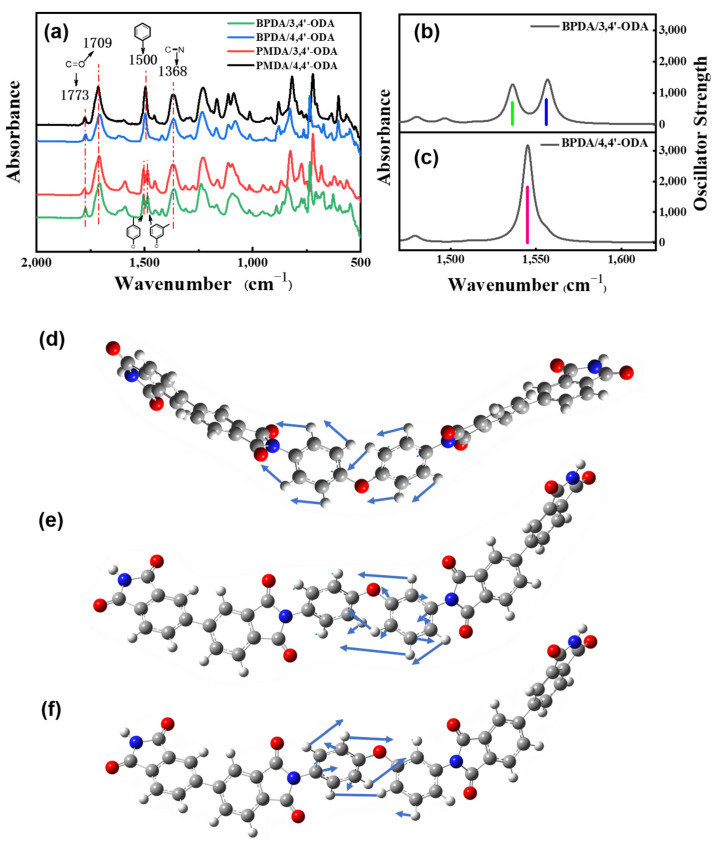
(**a**) The FT-IR curves of PI films; (**b**) the FT-IR simulation curve of BPDA/3,4′-ODA; (**c**) the FT-IR simulation curve of BPDA/4,4′-ODA; (**d**–**f**) the vibration trajectory of the functional group.

The chemical structure of the PI films characterized by FT-IR is shown in Figure 3a. We found three characteristic absorption bands of polyimide at 1773 cm^−1^, 1709 cm^−1^, and 1368 cm^−1^, which are attributed to the C-O asymmetrical stretching of imide groups, C-O symmetrical stretching of imide groups, and C-N stretching of the imide ring. The characteristic absorption bands at 1660 cm^−1^ and 1550 cm^−1^ of the precursor PAA did not appear at all, indicating that our films are completely cyclized into polyimide.

Interestingly, the peaks of the benzene ring of BPDA/3,4′-ODA and PMDA/3,4′-ODA around 1500 cm^−1^ are not single peaks. The FT-IR of these systems was simulated through Gaussian simulation, and we show the results taking the BPDA systems as examples. Figure 3b is the FT-IR simulation curve of BPDA/3,4′-ODA, and Figure 3c is the FT-IR simulation curve of BPDA/4,4′-ODA. The oscillator strength of the two peaks in Figure 3b is 351 (left) and 450 (right), and the peak intensity in Figure 3c is 912 cm^−1^. Figure 3d is the vibration trajectory of the functional groups belonging to BPDA/4,4′-ODA. Figure 3e,f is the vibration trajectory of the functional groups belonging to BPDA/3,4′-ODA when the wavenumber is larger and smaller, respectively. It can be observed that the higher wavenumber is the para-substituted peak of the benzene ring, and the lower wavenumber is the meta-substituted peak of the benzene ring. It indicates that the synthesis of polyimide material was successful.

### 3.2. Analysis of Molecular Chain Conformation

**Figure 4:**
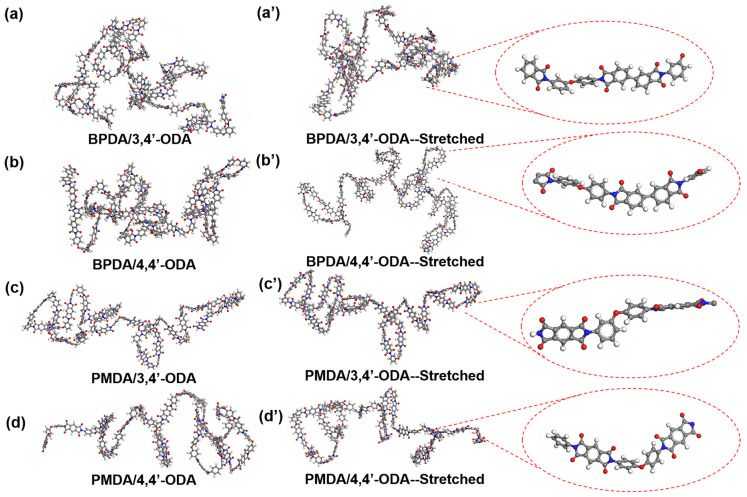
Molecular chain conformations of each system before (**a**–**d**) and after stretching (**a**′–**d**′).

In order to compare the deformation resistance and the changes of the internal microstructure of the material under the same stress, the molecular chain was extracted from the cell after 1000 ps NVT MD equilibrium of each PI system as the initial conformation of the PI molecular chain of the system. Then again, the molecular chain was extracted from the cell after applying 8 GPa stress in the same direction of each system as the stretching conformation of the PI molecular chain of the system, as shown in Figure 4.

It can be observed that the molecular chains of BPDA/3,4′-ODA in Figure 4a′ and BPDA/4,4′-ODA systems in Figure 4b′ are folded. The molecular chains of PMDA/3,4′-ODA in Figure 4c′ systems are also curled without stretching, but some segments are straight chains, and it is easier to form a chain orientation. After stretching, the proportion of straight chains is higher. The molecular chains of PMDA/4,4′-ODA systems in Figure 4d′ are spring-like spirals; it is difficult to have a relatively regular structure in some parts, and it is difficult to form a chain orientation and a molecular chain orientation. After careful analysis of the molecular chain conformation before and after stretching, it is observed that the molecular chains of the BPDA/3,4′-ODA system and the BPDA/4,4′-ODA system have a certain probability of straight parts in the short range. However, due to the asymmetry of the BPDA monomer, it is difficult to form a real regular molecular chain. However, because of the symmetry of the PMDA monomer, the PMDA/3,4′-ODA system can form a ladder-like regular structure, which leads to short-range order and easy to form a regular molecular chain. The PMDA/4,4′-ODA system is difficult to form an ordered structure because the bond angle of the C-O-C chemical bond naturally forms a relatively curled structure. The order of the molecular chain will also affect the rigidity of the polymer and also can explain the PMDA/3,4′-ODA and PMDA/4,4′-ODA system, although it also contains a PMDA monomer, which has a similar chemical structure; however, the PMDA/3,4′-ODA system can obtain higher modulus, the PMDA/4,4′-ODA system modulus is relatively minimum, and the BPDA/3,4′-ODA and BPDA/4,4′-ODA modulus are in the middle of the two.

The calculated mean-square end-to-end distance <Ree^2^> and the mean-square radius of gyration <S^2^> of each system before and after stretching are listed in Table 1. We also calculated the <Ree^2^> of each chain fully straightened without destroying its molecular structure, and the results are shown in Table 1. The <Ree^2^>/<S^2^> of the PMDA/4,4′ -ODA system is the largest but has the lowest elastic modulus, indicating that the modulus is not directly related to <Ree^2^>/<S^2^> and molecular chain rigidity. By calculating <S^2^> -stretched/<S^2^>, we found that the change range of the cluster size of the PMDA/3,4′-ODA system was much smaller than that of the PMDA/4,4′-ODA system under the same strain. This indicates that the deformation resistance of the PMDA/3,4′-ODA system is significantly better than that of PMDA/4,4′-ODA in the length scale of wire cluster size. The <S^2^> -stretched/<S^2^> of BPDA/3,4′-ODA system is slightly smaller than that of BPDA/4,4′-ODA system, which is also the same as the modulus law of these systems.

**Figure 5:**
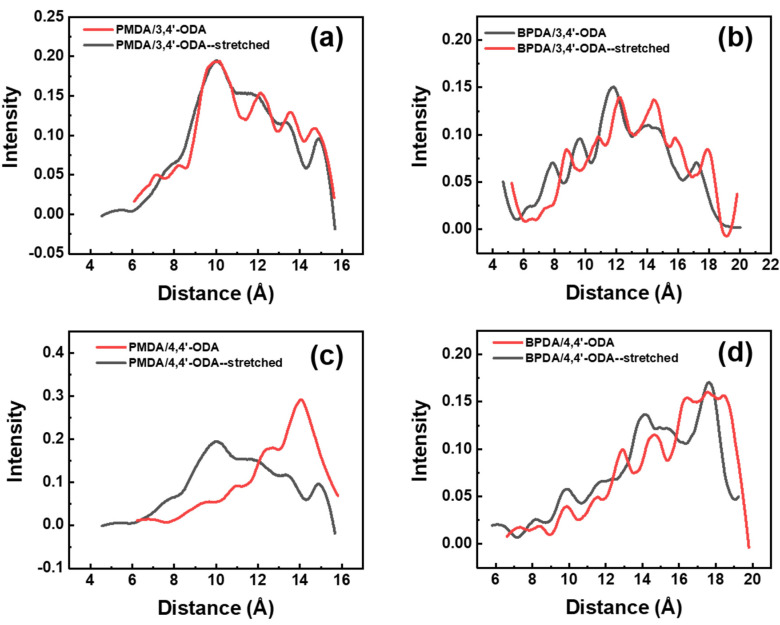
The distribution of a repeat unit length before and after stretching of different systems of PI. (**a**) PMDA/3,4′-ODA; (**b**) BPDA/3,4′-ODA; (**c**) PMDA/4,4′-ODA; (**d**) BPDA/4,4′-ODA.

After analyzing the deformation resistance of each system at the scale of the molecular chain line, it was necessary to analyze the deformation resistance of each system at a smaller scale. The length of each repeating unit was statistically analyzed by MS before and after stretching. The distribution of repeating unit lengths was plotted into probability curves and noise reduction, and the result is shown in Figure 5. Through analysis of the curve, we found that the length of the repeat unit in the 3,4′-ODA system has little change from Figure 5a. There are slight peaks at 11.5 Å, 13.5 Å, and 14.5 Å, and the curve decreases at 11 Å. This fully illustrates that the 3,4′-ODA system has good deformation resistance under 8 GPa stress. The length of amorphous repeat units is largely located near 10 Å, and a large number of repeat units are still located near 10 Å under strain. The length of repeating units in the 4,4′-ODA system has changed greatly, from a large distribution near 10 Å to a concentrated distribution near 12 Å–14 Å and a strong peak at 14 Å. This shows that in the system under the action of stress, deformation occurred in a large number of repeating units, showing a very poor ability to resist deformation. Because the BPDA monomer itself is relatively long, the average length of the repeating unit of the system containing the BPDA monomer is longer than that of the system containing the PMDA monomer. For BPDA/3,4′-ODA, the curve moves to the right as a whole, forming new peaks at 14 Å, 16 Å, and 18 Å, but the overall distribution does not change significantly, which also reflects good deformation resistance. For the BPDA/4,4′-ODA system, it can be observed from the curve that the length of the repeat unit increases greatly between 16 Å and 19 Å, and the curve shifts to the right as a whole. It can be observed from the comparison that the system containing 3,4′-ODA has strong deformation resistance, and the system containing 4,4′-ODA has poor deformation resistance. Among them, the PMDA/3,4′-ODA system has the strongest deformation resistance, and the PMDA/4,4′-ODA system has the worst resistance. This result is consistent with the test results in the experiment, reflecting the rationality of the model. More importantly, a method for comparing the modulus of polymer is established, i.e., a method for qualitative analysis of the PI modulus and the variation of the conformation of molecular chains and repeated units under strain in the range of elastic deformation.

**Figure 6:**
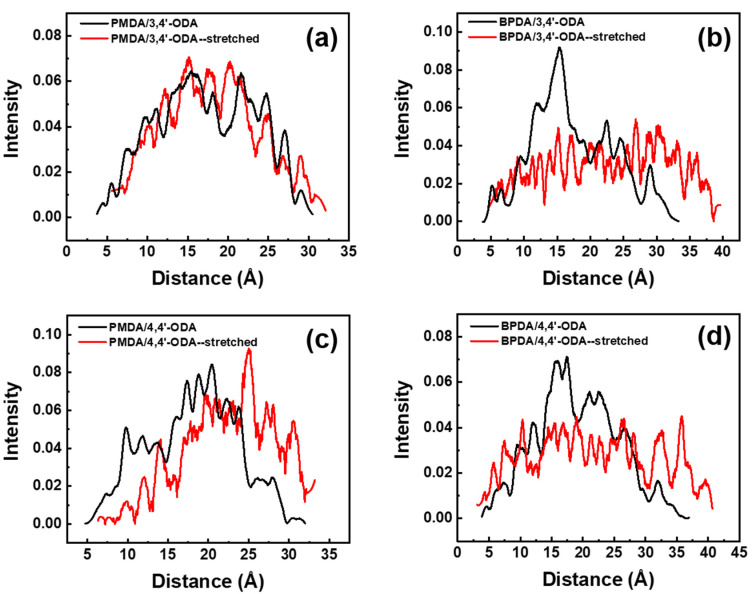
The distribution of two repeat unit lengths before and after stretching of different systems of PI. (**a**) PMDA/3,4′-ODA; (**b**) BPDA/3,4′-ODA; (**c**) PMDA/4,4′-ODA; (**d**) BPDA/4,4′-ODA.

The deformation resistance at the length scale of the two repeat units is shown in Figure 6. For the PMDA system, it can still be observed that the deformation resistance of the PMDA/3,4′-ODA system is better than that of the PMDA/4,4′-ODA system. However, the strength of the deformation resistance of BPDA system cannot be clearly compared under more than two repeat unit lengths, so it does not continue to expand the statistical scale.

From the above analysis, it can be concluded that the deformation resistance of the PMDA/3,4′-ODA system is significantly better than that of the PMDA/4,4′-ODA system based on the structural variation analysis from one repeating unit, two repeating units, and molecular chain scale. Similarly, the deformation resistance of the BPDA/3,4′-ODA system is slightly better than that of BPDA/4,4′-ODA.

### 3.3. Mechanism Analysis

For material deformation, there are four influencing factors: intermolecular forces, the change of covalent bond length, the change of internal rotation angle, and the change of bond angle. Because the deformation of each tensile model is in the range of elastic deformation, no molecular chain slip occurs, so the intermolecular force is not discussed.

**Figure 7:**
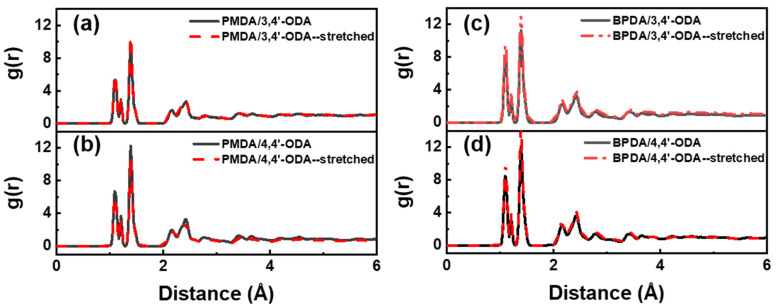
All-atom RDF of the systems before and after stretching. (**a**) PMDA/3,4′-ODA; (**b**) BPDA/3,4′-ODA; (**c**) PMDA/4,4′-ODA; (**d**) BPDA/4,4′-ODA.

To discuss the changes of the covalent bond length before and after stretching, the covalent bond changes in the PI of each system were analyzed by all-atom RDF. This method is to count the probability of atoms appearing at a certain distance from an atom, which might be used to reflect the bond length. The results are shown in Figure 7. The peaks at 1.1 Å and 1.2 Å are the bond lengths of C-H and C=O, respectively. The peak at 1.4 Å is very strong, and the composition is more complex, consisting of C-N on the imide ring, C-C on the benzene ring, and C-O in two kinds of ODA. Through comparison, it is found that the RDF curve changes before and after stretching are very small, indicating that the change in PI covalent bond length before and after stretching is negligible, which is not the main reason for the material modulus.

**Figure 8:**
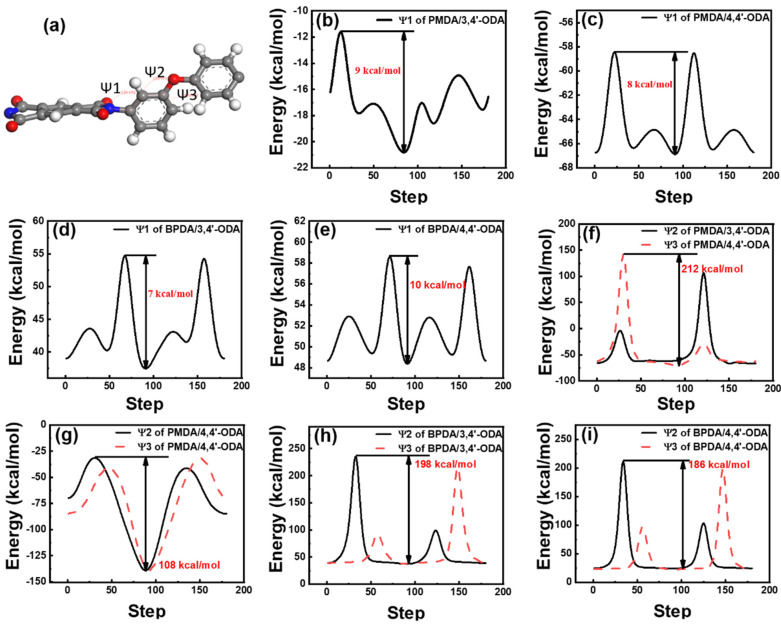
Torsional angular barrier curves of different systems of PI. (**a**) Ψ1, Ψ2, Ψ3 of repeat unit; (**b**) Ψ1 of PMDA/3,4′-ODA; (**c**) Ψ2 of PMDA/4,4′-ODA; (**d**) Ψ1 of BPDA/3,4′-ODA; (**e**) Ψ1 of BPDA/4,4′-ODA; (**f**) Ψ2 and Ψ3 of PMDA/3,4′-ODA; (**g**) Ψ2 and Ψ3 of PMDA/4,4′-ODA; (**h**) Ψ2 and Ψ3 of BPDA/3,4′-ODA; (**i**) Ψ2 and Ψ3 of BPDA/4,4′-ODA.

The difference in the chemical structure affects the conformation of the repeating unit. The internal rotation angle energy barrier of the repeating unit is obtained by MS. The rotation barriers of the internal rotation angle Ψ1, Ψ2, and Ψ3 of these systems are shown in Figure 8. By comparison, it is observed that the Ψ1′s energy barrier of each system has little fluctuation and is much smaller than Ψ2 and Ψ3, which is roughly distributed between 7 kcal/mol and 10 kcal/mol. Thus, the modulus provided by Ψ1 is almost the same. The PMDA/4,4′-ODA system has the smallest Ψ2′s and Ψ3′s rotation barriers of 108 kcal/mol, and the PMDA/3,4′-ODA system has the largest Ψ2′s and Ψ3′s rotation barriers of 212 kcal/mol. The order of Ψ2′s and Ψ3′s rotation barriers from big to small is PMDA/3,4′-ODA>BPDA/3,4′-ODA>BPDA/4,4′-ODA>PMDA/4,4′-ODA; it is consistent with the order of the modulus, thus indicating that the internal rotation angle rotation barrier is the key factor affecting the modulus of the PI film. This indicates that the modulus provided by the internal rotation of ether bonds in each system is quite different, which is a key factor affecting the mechanical properties of each system.

**Figure 9:**
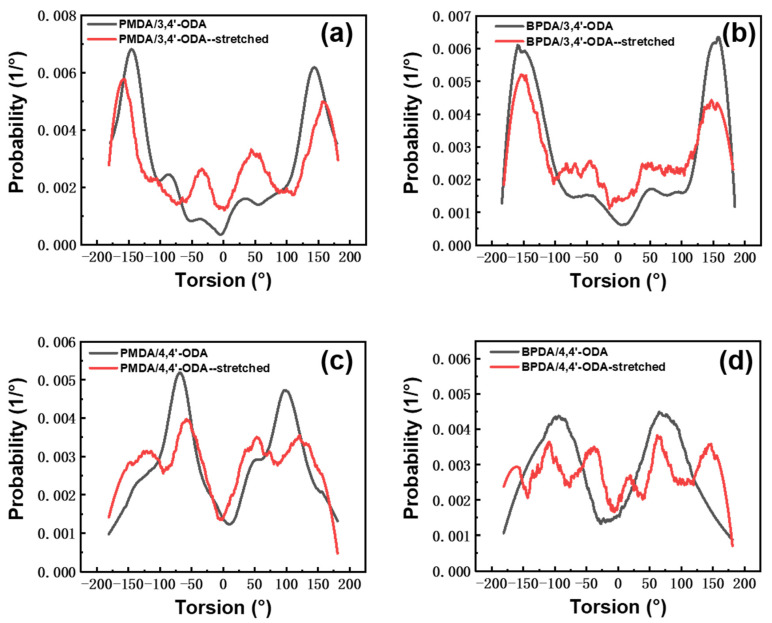
Torsion angles analysis of different systems of PI. (**a**) PMDA/3,4′-ODA; (**b**) BPDA/3,4′-ODA; (**c**) PMDA/4,4′-ODA; (**d**) BPDA/4,4′-ODA.

Through MS, the torsion angle changes of Ψ3 in each system before and after stretching were counted, the curve of torsion angle distribution and probability was drawn, and the noise was reduced. The result is shown in Figure 9. Compared with the PMDA system and BPDA system, it is obvious that the torsional angle of the system containing 3,4′-ODA changes less, and the torsional angle of the system containing 4,4′-ODA changes more. During stretching, the torsional angles of PMDA/3,4′-ODA system at −145° and 145° decreased slightly, and the peak values moved to −158° and 158°. The torsional angles at −35° and 35° increased correspondingly, but more torsional angles remained near the original angle. For the PMDA/4,4′-ODA system, the peaks at −70° and 100° decreased significantly, and this conformation disappeared in large quantities and transformed into the conformations with torsion angles of −155° and 55°. This reflects that the torsional angle of the PMDA/3,4′-ODA system has changed in the deformation process. However, due to its high torsional energy barrier, it needs to absorb a large amount of energy to complete the torsion. The total number of changed conformations is significantly less than that of PMDA/4,4′-ODA, which is shown as a high modulus in macroscopic properties. The law of the BPDA system is similar to that of the PMDA system. For the BPDA/3,4′-ODA system, the peaks at −157° and 158° slightly weaken and move to −153° and 145°, and the peaks near −47° and 47° slightly increase. For the BPDA/4,4′-ODA system, the peaks near −95° and 70° on the curve decrease significantly, and become multiple peaks at −161°, −109°, −37°, 19°, 63°, and 146°, indicating that more conformations have changed. Compared with BPDA/3,4′-ODA, the conformational changes of the BPDA/3,4′-ODA system are more and more obvious, and the macroscopic performance is that the modulus is lower than that of the BPDA/3,4′-ODA system.

**Figure 10:**
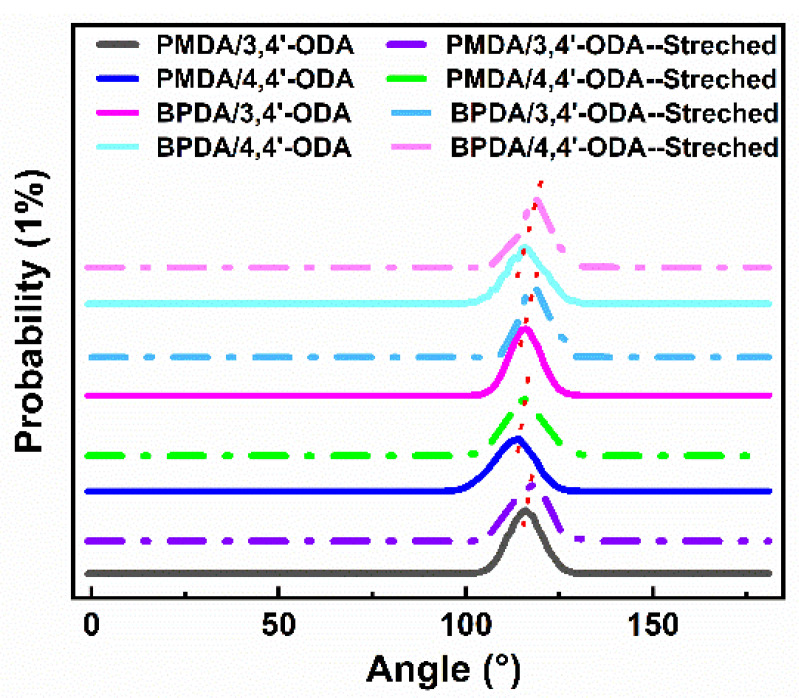
Angle changes of C-O-C in ODA before and after stretching of different systems of PI.

There is a wide range of C-O-C bond angles. In order to study the change of bond angle before and after stretching, the C-O-C bond angle before and after stretching was counted, as shown in Figure 10. It can be observed that during the stretching process, the bond angle of each system increases slightly, and the peak in the figure shifts about 1.5° to the right. However, this change is far from enough to double the deformation of the polymer.

### 3.4. Thermal Performance Analysis

**Figure 11:**
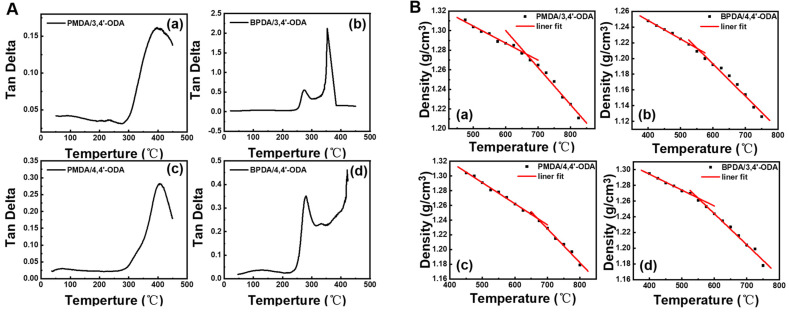
(**A**) The DMA curves of different systems of PI; (**B**) Tgs of PI obtained by simulation.

For polyimide film, its thermal property is an important indicator for engineering applications. The Tgs of these systems were tested through the DMA test. The curves of the DMA are shown in Figure 11A, and the test results of Tgs are listed in Table 2. The results showed that the orders of calculated Tgs are PMDA/4,4′-ODA (406 °C) > PMDA/3,4′-ODA (396 °C) > BPDA/4,4′-ODA (281 °C) > BPDA/3,4′-ODA (274 °C).

To calculate Tgs, we performed 500 ps NPT and NVT MD simulations at different temperatures, then the density-temperature relationship of polyimides obtained at different temperatures was depicted in Figure 11B, and the Tgs can be obtained from the inflection point of the fitting curves. It can be seen from Table 2 that the calculated values are relatively close to the test results, which further illustrates the rationality of the models. Tgs can usually reflect the rigidity and mobility of the molecular chains. The results showed that the orders of calculated Tgs are PMDA/4,4′-ODA > PMDA/3,4′-ODA > BPDA/4,4′-ODA ≈ BPDA/3,4′-ODA.

**Figure 12:**
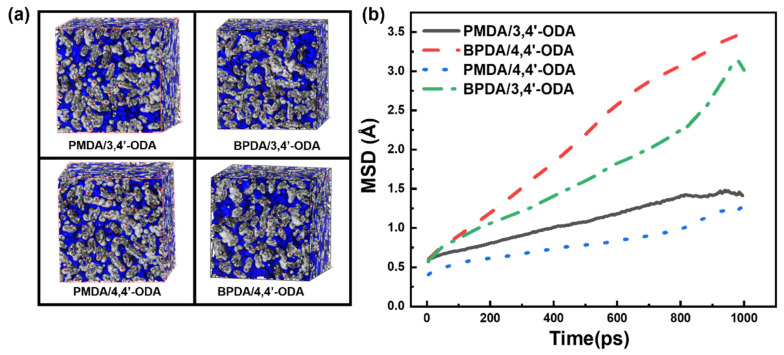
(**a**) The FFV of different PI models; (**b**) the MSD curves of different PI models.

The FFV of each system calculated by MS is shown in Figure 12a. The density, specific volume, van der Waals volume, and FFV of each system are listed in Table 2. It can be observed that the FFV from large to small is BPDA/4,4′-ODA > BPDA/3,4′-ODA > PMDA/3,4′-ODA > PMDA/4,4′-ODA. This law and Tg from low to high has been, according to the free volume theory, that the greater the FFV, the more cavities can provide the motion of the chain, so there will be lower Tg. The mean-square displacement (MSD) of each system was calculated to analyze the dynamic motion ability of the molecular chain. The MSD curves are shown in Figure 12b. The higher the MSD curve is, the better the motion ability of the polymer molecular chain. The MD simulation of the PI molecular chain at 298 K for 1000 ps was carried out. By comparison, we found that the stronger the molecular chain movement ability, the lower the Tg.

## 4. Conclusions

The purpose of the current research was to determine factors that have the greatest impact on modulus after chemical structure changes. Analysis of the change of covalent bond length, bond angle, and torsion angle clearly indicates that the rotation in molecular chains is a key factor in providing modulus. What is more, by intuitively comparing the deformation resistance of different PIs systems at different length scales under the same strain, i.e., one repeating unit, two repeating units, and the whole molecular chain length scale, we have developed an efficient method in the present work to qualitatively predict the order of the modulus of the polymers. Both the experimental test and MD simulation show that the PI of PMDA/3,4′-ODA has high Tg and good deformation resistance, namely a high modulus. This PI itself has short-range rigid-straight units, and the molecular chain is straighter after stretching, which can be more neatly arranged, accounting for its better mechanical properties. Further research should be undertaken to exert the potential of this structure to prepare polyimide materials with long-range ordered structures such as polyimide fibers.

## Figures and Tables

**Table 1:** <Ree^2^> and <S^2^> before and after stretching.

Systems	<Ree^2^>/Å^3^	<S^2^>/Å^3^	<Ree^2^>/<S^2^>	<S^2^>-Stretched/Å^3^	<Ree^2^>-Stretched/Å^3^	<S^2^>-Stretched/<S^2^>
PMDA/3,4′-ODA	3015	426	7.07	446	3150	1.047
PMDA/4,4′-ODA	3603	480	7.50	543	3817	1.131
BPDA/3,4′-ODA	3309	462	7.16	488	3326	1.056
BPDA/4,4′-ODA	3485	573	6.08	625	3782	1.091

**Table 2:** Tgs, density, specific volume, Van der Waals volume, and FFV of different systems of PIs.

Systems	Tg-Experimental (°C)	Tg-Simulation (°C)	Density (g/cm^3^)	Specific Volume (Å^3^)	Van der Waals Volume (Å^3^)	FFV
BPDA/4,4′-ODA	281	277	1.297	77.072	42.675	0.280
BPDA/3,4′-ODA	274	276	1.306	76.518	42.666	0.275
PMDA/4,4′-ODA	406	397	1.436	69.636	41.139	0.232
PMDA/3,4′-ODA	396	391	1.321	75.705	42.666	0.267

## Data Availability

The data presented in this study are available on request from the corresponding author.

## References

[1] Ni H., Liu J., Wang Z., Yang S. (2015). A Review on Colorless and Optically Transparent Polyimide Films: Chemistry, Process and Engineering Applications. J. Ind. Eng. Chem..

[2] Liaw D.-J., Wang K.-L., Huang Y.-C., Lee K.-R., Lai J.-Y., Ha C.-S. (2012). Advanced Polyimide Materials: Syntheses, Physical Properties and Applications. Prog. Polym. Sci..

[3] Ding Y., Hou H., Zhao Y., Zhu Z., Fong H. (2016). Electrospun Polyimide Nanofibers and Their Applications. Prog. Polym. Sci..

[4] Vanherck K., Koeckelberghs G., Vankelecom I.F.J. (2013). Crosslinking Polyimides for Membrane Applications: A Review. Prog. Polym. Sci..

[5] Zhou Z., Zhang Y., Liu S., Chi Z., Chen X., Xu J. (2016). Flexible and Highly Fluorescent Aromatic Polyimide: Design, Synthesis, Properties, and Mechanism. J. Mater. Chem. C.

[6] Choi M.-C., Kim Y., Ha C.-S. (2008). Polymers for Flexible Displays: From Material Selection to Device Applications. Prog. Polym. Sci..

[7] Gan F., Dong J., Tang M., Li X., Li M., Zhao X., Zhang Q. (2019). High-Tenacity and High-Modulus Polyimide Fibers Containing Benzimidazole and Pyrimidine Units. React. Funct. Polym..

[8] Cheng S.Z.D., Wu Z., Mark E., Steven L.C.H., Frank W.H. (1991). A High-Performance Aromatic Polyimide Fibre: 1. Structure, Properties and Mechanical-History Dependence. Polymer.

[9] Wen P., He R., Li X.-D., Lee M.-H. (2017). Syntheses and Characterizations of High Refractive Index and Low Birefringence Polyimides Containing Spirobifluorene in the Side Chain. Polymer.

[10] Lian R., Lei X., Chen Y., Zhang Q. (2019). Hyperbranched-Polysiloxane-Based Hyperbranched Polyimide Films with Low Dielectric Permittivity and High Mechanical and Thermal Properties. J. Appl. Polym. Sci..

[11] Ma S., Wang S., Jin S., Wang Y., Yao J., Zhao X., Chen C. (2020). Construction of High-Performance, High-Temperature Shape Memory Polyimides Bearing Pyridine and Trifluoromethyl Group. Polymer.

[12] Deng B., Zhang S., Liu C., Li W., Zhang X., Wei H., Gong C. (2018). Synthesis and Properties of Soluble Aromatic Polyimides from Novel 4,5-Diazafluorene-Containing Dianhydride. RSC Adv..

[13] Wang X., Wang H., Luo L., Huang J., Gao J., Liu X. (2012). Dependence of Pretilt Angle on Orientation and Conformation of Side Chain with Different Chemical Structure in Polyimide Film Surface. RSC Adv..

[14] Zhang M., Liu C., Yang J., Yang P., Zhang L., Dong J. (2017). Analysis of the Herbicidal Mechanism of 4-Hydroxy-3-Methoxy Cinnamic Acid Ethyl Ester Using ITRAQ and Real-Time PCR. J. Proteom..

[15] Jain A., Ong S.P., Hautier G., Chen W., Richards W.D., Dacek S., Cholia S., Gunter D., Skinner D., Ceder G. (2013). Commentary: The Materials Project: A Materials Genome Approach to Accelerating Materials Innovation. APL Mater..

[16] Sun H. (1998). COMPASS:  An Ab Initio Force-Field Optimized for Condensed-Phase ApplicationsOverview with Details on Alkane and Benzene Compounds. J. Phys. Chem. B.

[17] Van Gunsteren W.F., Daura X., Hansen N., Mark A.E., Oostenbrink C., Riniker S., Smith L.J. (2018). Validation of Molecular Simulation: An Overview of Issues. Angew. Chem. Intern. Ed..

[18] Calle M., García C., Lozano A.E., de la Campa J.G., de Abajo J., Álvarez C. (2013). Local Chain Mobility Dependence on Molecular Structure in Polyimides with Bulky Side Groups: Correlation with Gas Separation Properties. J. Membr. Sci..

[19] Swaidan R., Ghanem B., Al-Saeedi M., Litwiller E., Pinnau I. (2014). Role of Intrachain Rigidity in the Plasticization of Intrinsically Microporous Triptycene-Based Polyimide Membranes in Mixed-Gas CO_2_/CH_4_ Separations. Macromolecules.

[20] Tan P.C., Ooi B.S., Ahmad A.L., Low S.C. (2018). Monomer Atomic Configuration as Key Feature in Governing the Gas Transport Behaviors of Polyimide Membrane. J. Appl. Polym. Sci..

[21] Swaidan R., Ghanem B., Litwiller E., Pinnau I. (2015). Effects of Hydroxyl-Functionalization and Sub-Tg Thermal Annealing on High Pressure Pure- and Mixed-Gas CO_2_/CH_4_ Separation by Polyimide Membranes Based on 6FDA and Triptycene-Containing Dianhydrides. J. Membr. Sci..

[22] Tanis I., Brown D., Neyertz S.J., Heck R., Mercier R. (2014). A Comparison of Homopolymer and Block Copolymer Structure in 6FDA-Based Polyimides. Phys. Chem. Chem. Phys..

[23] Lei H., Qi S., Wu D. (2019). Hierarchical Multiscale Analysis of Polyimide Films by Molecular Dynamics Simulation: Investigation of Thermo-Mechanical Properties. Polymer.

[24] Li Y., Zhang M., Han E., Zhu L., Xiao M., Huanyu L., Niu H., Wu D. (2020). Effect of Introduction of Fluoromonomer Copolymerization on Properties of Polyimide Hollow Fibers. High Perform. Polym..

[25] Lin D., Jiang M., Qi S., Wu D. (2020). Macromolecular Structural Evolution of Polyimide Chains during Large-Ratio Uniaxial Fiber Orientation Process Revealed by Molecular Dynamics Simulation. Chem. Phys. Lett..

[26] Nazarychev V.M., Lyulin A.V., Larin S.V., Gofman I.V., Kenny J.M., Lyulin S.V. (2016). Correlation between the High-Temperature Local Mobility of Heterocyclic Polyimides and Their Mechanical Properties. Macromolecules.

[27] Takeichi T., Takenoshita H., Ogura S., Inagaki M. (1994). Carbonization of Polyimide Films: Effect of Cold-Drawing and Chemical Structure. J. Appl. Polym. Sci..

[28] Su J.-F., Chen L., Tang T.-T., Ren C.-B., Wang J.-J., Qin C.-X., Dai L.-X. (2011). Preparation and Characterization of Ternary Copolyimide Fibers via Partly Imidized Method. High Perform. Polym..

[29] Liu X., Gao G., Dong L., Ye G., Gu Y. (2009). Correlation between Hydrogen-Bonding Interaction and Mechanical Properties of Polyimide Fibers. Polym. Adv. Technol..

[30] Pan R., Liu X., Zhang A., Gu Y. (2007). Molecular Simulation on Structure–Property Relationship of Polyimides with Methylene Spacing Groups in Biphenyl Side Chain. Comput. Mater. Sci..

[31] Goyal S., Park H.-H., Lee S.H., Savoy E., McKenzie M.E., Rammohan A.R., Mauro J.C., Kim H., Min K., Cho E. (2016). Characterizing the Fundamental Adhesion of Polyimide Monomers on Crystalline and Glassy Silica Surfaces: A Molecular Dynamics Study. J. Phys. Chem. C.

[32] Low B.T., Xiao Y., Chung T.S. (2009). Amplifying the Molecular Sieving Capability of Polyimide Membranes via Coupling of Diamine Networking and Molecular Architecture. Polymer.

[33] Chang K.-S., Tung C.-C., Wang K.-S., Tung K.-L. (2009). Free Volume Analysis and Gas Transport Mechanisms of Aromatic Polyimide Membranes: A Molecular Simulation Study. J. Phys. Chem. B.

[34] Heuchel M., Hofmann D. (2002). Molecular Modelling of Polyimide Membranes for Gas Separation. Desalination.

[35] Zhang L., Xiao Y., Chung T.-S., Jiang J. (2010). Mechanistic Understanding of CO_2_-Induced Plasticization of a Polyimide Membrane: A Combination of Experiment and Simulation Study. Polymer.

[36] Chang K.-S., Wu Z.-C., Kim S., Tung K.-L., Lee Y.M., Lin Y.-F., Lai J.-Y. (2014). Molecular Modeling of Poly (Benzoxazole-Co-Imide) Membranes: A Structure Characterization and Performance Investigation. J. Membr. Sci..

[37] Gissinger J.R., Pramanik C., Newcomb B., Kumar S., Heinz H. (2018). Nanoscale Structure—Property Relationships of Polyacrylonitrile/CNT Composites as a Function of Polymer Crystallinity and CNT Diameter. ACS Appl. Mater. Interfaces.

[38] Lyulin A.V., Balabaev N.K., Michels M.A.J. (2003). Molecular-Weight and Cooling-Rate Dependence of Simulated Tg for Amorphous Polystyrene. Macromolecules.

[39] Velioğlu S., Ahunbay M.G., Tantekin-Ersolmaz S.B. (2012). Investigation of CO_2_-Induced Plasticization in Fluorinated Polyimide Membranes via Molecular Simulation. J. Membr. Sci..

[40] Park C.H., Tocci E., Kim S., Kumar A., Lee Y.M., Drioli E. (2014). A Simulation Study on OH-Containing Polyimide (HPI) and Thermally Rearranged Polybenzoxazoles (TR-PBO): Relationship between Gas Transport Properties and Free Volume Morphology. J. Phys. Chem. B.

[41] Nazarychev V.M., Larin S.V., Yakimansky A.V., Lukasheva N.V., Gurtovenko A.A., Gofman I.V., Yudin V.E., Svetlichnyi V.M., Kenny J.M., Lyulin S.V. (2015). Parameterization of Electrostatic Interactions for Molecular Dynamics Simulations of Heterocyclic Polymers. J. Polym. Sci. Part B Polym. Phys..

